# The seroprevalence of hepatitis E virus infection in the preselected population from Romania: a hospital survey

**DOI:** 10.1186/1471-2334-14-S7-O28

**Published:** 2014-10-15

**Authors:** Valeriu Gheorghiță, Anca Streinu-Cercel, Oana Săndulescu, Mădălina Popa, Simona Elena Albu, Florin Alexandru Căruntu, Adrian Streinu-Cercel

**Affiliations:** 1 National Institute for Infectious Diseases "Prof. Dr. Matei Balş", Bucharest, Romania; 2 Central Universitary Emergency Military Hospital Dr Carol Davila, Bucharest, Romania; 3 Carol Davila University of Medicine and Pharmacy, Bucharest, Romania; 4 University Emergency Hospital of Bucharest, Romania

## Background

Hepatitis E virus (HEV) infection is a significant public health problem in many parts of the world. The virus is classified into four major genotypes, representing a single serotype. HEV genotypes 1 and 2 are restricted to humans and usually transmitted via fecally-contaminated water, resulting in large outbreaks and epidemics of acute hepatitis in developing countries. HEV genotypes 3 and 4 can infect humans as well as other mammalian species (e.g. pigs) and associate sporadic cases of autochthonous hepatitis E, recently described in developed countries. In these areas, zoonotic transmission appears to play a major role. Moreover, genotype 3 HEV infection can cause chronic hepatitis in immunosuppressed patients. The estimated seroprevalence of HEV infection in these regions is variable, between less than 5% and 52% in southwestern France. To date, no HEV seroprevalence studies have been performed in Romania.

## Study hypothesis

The specific aim of this observational, cross-sectional study is to estimate the prevalence of serum anti-HEV IgG antibodies in the Romanian population. The study population will include 1000 consecutive patients admitted in two medical centers from Bucharest over a period of one year: 900 patients in the National Institute for Infectious Diseases "Prof. Dr. Matei Balş" and 100 female patients in the Department of Gynecology of the University Emergency Hospital, Bucharest. The main inclusion criteria are obtaining the informed consent and the age over 18 years. Additionally for gynecology, the female patients must be pregnant. The blood tests will focus on determination of HEV IgG antibodies using ELISA tests. Each sample must be accompanied by an additional questionnaire completed by the patient.

## Expected Results

Being a country with many patients living in rural areas with predominantly agricultural occupation, we anticipate finding a variable rate of HEV seroprevalence according to different parameters: age, daily activity (e.g. pig farmer), rural area residency.

## Conclusion

The discovery of autochthonous cases of hepatitis E in developed countries has substantially changed our understanding of HEV infection. As part of the European Union and considering the data already reported by many European countries, we need to determine the HEV IgG seroprevalence in our country, in order to be able to develop, if necessary, certain public health policies for decreasing both the human HEV transmission and the morbidity related to HEV infection.

